# Recent advances in understanding provoked vestibulodynia

**DOI:** 10.12688/f1000research.9603.1

**Published:** 2016-10-26

**Authors:** Ahinoam Lev-Sagie, Steven S. Witkin

**Affiliations:** 1 Department of Obstetrics and Gynecology, Hadassah-Hebrew University Medical Center, Jerusalem, Israel; 2 Department of Obstetrics and Gynecology, Weill Cornell Medicine, New York, NY, USA

**Keywords:** Vulvodynia, vulvar pain syndromes, pain mechanisms

## Abstract

Vulvodynia refers to pain in the vulva of at least 3 months’ duration in the absence of a recognized underlying cause. Provoked, localized vestibulodynia is the term used to describe superficial pain confined to the vulvar vestibule, provoked by touch. This review will focus on provoked vestibulodynia with regard to its suggested causative factors and will discuss the role of inflammation, vulvovaginal infections, mucosal nerve fiber proliferation, hormonal associations, central pain mechanisms, pelvic floor muscle dysfunction, and genetic factors.

Clinical observations, epidemiological studies, and data from basic research emphasize the heterogeneity of vulvar pain syndromes. There is a critical need to perform prospective, longitudinal studies that will allow better diagnostic criteria and subgrouping of patients that would lead to improvements in our understanding of provoked vestibulodynia and its treatment.

## Introduction

Vulvodynia is a diagnosis of exclusion. It refers to ‘pain in the vulva’ of at least 3 months’ duration, in the absence of a recognized underlying cause, and can consist of various clinical features. Some women with this diagnosis have continuous, diffuse vulvar pain (generalized, unprovoked vulvodynia), while others experience localized pain, usually provoked by touch. The current nomenclature ^1^ uses a symptom-based classification to characterize the pain with regard to its location (localized, generalized, or mixed), the conditions that provoke it (contact, spontaneous, or mixed), its temporal pattern (intermittent or constant), and its onset (primary or secondary).

The vulvar pain syndrome currently known as provoked, localized vestibulodynia (PVD) is the term used to describe superficial pain confined to the vulvar vestibule, provoked by touch. Some researchers suspect that PVD and generalized, unprovoked vulvodynia may represent a continuum of the same condition ^2^. However, owing to its separate clinical presentation, this communication will focus only on PVD. In some women, their vestibular pain can be caused by minimal touch (sitting or tight-fitting clothing), whereas in others it is provoked only by vaginal penetration during sexual intercourse, tampon insertion, or gynecological examination, resulting in dyspareunia or a complete inability to have intercourse.

Some women have a primary form of PVD (PVD1), experiencing pain at first introital touch, while others describe a period of pain-free vaginal penetration before the onset of symptoms and are defined as having secondary PVD (PVD2). The pain is often described as a burning or cutting sensation and may be located throughout the vestibule or confined to the lower vestibule. Erythema may or may not be found and is no longer considered a defining criterion.

No single causative factor of PVD has yet been identified, and its etiology is considered multifactorial. The accepted theory is that PVD represents a diverse group of disorders, causing similar symptoms. The clinical diagnosis of PVD is defined by subjective signs and symptoms of entry dyspareunia and vestibular tenderness to gentle touch after the exclusion of defined disorders (infection, dermatosis, etc.). Current treatment strategies follow a “trial-and-error” approach, guided mainly by expert opinion and strategies used in other pain disorders ^3, 4^ rather than by an evidence-based approach from randomized clinical trials ^5^.

Much effort has been made to find pathophysiological changes characteristic of PVD. A range of abnormalities in different systems (the vestibular mucosa, pelvic floor musculature, and peripheral and central pain regulation) as well as in different pathways (inflammatory, hormonal, genetic, psychosocial, etc.) have been identified in groups of subjects. This review will focus on our current understanding of the suggested etiologies of PVD.

## Peripheral pain mechanisms

Hypersensitivity of the vulvar vestibule is one of the defining characteristics of PVD. Quantitative sensory testing of vestibular mucosa indicates peripheral sensitization to both mechanical and thermal stimuli ^6^. One hypothesis suggests that the enhancement of peripheral pain perception may be caused by changes in the biochemical environment, modifying the conduction of nerve fibers, further lowering the threshold in nociceptors ^2^. Another suggested mechanism is hyperinnervation of the vestibular mucosa. Increased vestibular nerve fiber density was first described by Westrom and Willen ^7^ and was thereafter confirmed by others ^8– 13^. Immunohistochemical methods defined these nerve endings as nociceptors ^14^. This hyperinnervation corresponds to heightened mechanical allodynia (pain from light touch) and hyperalgesia (enhanced pain perception) and is termed “neuroproliferation”. Neuroproliferation is hypothesized to be either congenital or acquired.

### Primary, secondary, acquired, and congenital neuroproliferative PVD

Leclair *et al*. ^13^ showed that PVD1 patients have significantly greater neural hypertrophy as compared to those with PVD2, supporting the hypothesis that PVD1 and PVD2 may have distinct histopathologic pathways rather than representing different stages of the same disease.

It is possible that PVD1 may be congenital, or that mucosal hypersensitivity may be acquired in early life, initially being recognized in adolescence. A correlation between early life adverse events and PVD in adults has been found in case-control studies ^15– 17^ and was attributed to dysfunctional regulation of the hypothalamic–pituitary–adrenal axis ^18^. Recent studies in animal models found evidence that early life stress in mice induced increased vaginal sensitivity ^18^ and that chemical neonatal vaginal irritation led to permanent vaginal hypersensitivity ^19^.

It was also suggested that PVD may be congenital in some women ^20, 21^. Embryologically, the vestibule is of endodermal origin, derived from the urogenital sinus, and neuroproliferation may represent a congenital anomaly. In such cases, the neural hypersensitivity is primary and may also be present in other tissues derived from the urogenital sinus. This may explain the coexistence of PVD and interstitial cystitis in some women ^22^, as well as the significantly higher level of umbilical sensitivity in women with PVD1 ^23^. Acquired PVD has also been attributed to neural proliferation in response to an inflammatory process (see below) or endocrine factors ^24, 25^.

The significance of nociceptor hypertrophy is controversial; some researchers consider neuroproliferation as a non-specific reaction to previous mucosal trauma or inflammation ^26^ and attribute the enhanced pain perception to neurogenic inflammation ^26^. In addition, and in contrast to what one might expect, it is well accepted that reduced, and not increased, intraepidermal nerve fiber density is associated with an elevated risk of developing neuropathic pain ^2, 27^. However, an additional observation supporting the contribution of peripheral pain mechanisms to PVD is that 80% of patients undergoing surgical excision (“vestibulectomy”), in which the hyperinnervated region is removed, experience symptomatic relief. However, it is important to recognize the lack of randomized trials and variation in the definition of successful outcome.

## Inflammatory mechanisms and vulvovaginal infections

PVD was initially regarded as a chronic local inflammatory condition and was therefore called “vulvar vestibulitis syndrome” ^28^. In vulvar biopsies obtained from PVD patients, the subepithelial part of the lamina propria is infiltrated by inflammatory cells, mainly T lymphocytes. This is often described as non-specific chronic inflammation ^29, 30^. However, in later studies, a similar inflammatory infiltrate was also found in the vestibule of healthy women ^12, 31^. Consequently, the initial inflammatory theory has been abandoned and the condition is now regarded as a pain syndrome ^32^.

Nevertheless, multiple studies suggest that inflammation may play a role in the development of PVD. The suggested mechanism hypothesizes that persistent inflammation in the vestibular mucosa promotes hyperplasia of nociceptive c-fibers, secondary to the production of nerve growth factor, altered receptor expression ^33^, and persistent elevation of pro-inflammatory substances ^8, 14, 34, 35^. These changes in the biochemical milieu could alter the ion channel activity of peripheral pre-terminal axons, which leads to a lowered mechanical, thermal, or chemical threshold in the primary nociceptors ^2^. Consequently, even light touch can result in exaggerated release of pro-inflammatory mediators by sensitized nerve fibers ^36^. This, in turn, activates neuroendocrine cells and mast cells to release additional pro-inflammatory compounds. This self-perpetuating process of neurogenic inflammation is thought to play a key role in the maintenance of local inflammation in PVD ^10, 37^.

Studies that have evaluated inflammatory characteristics in PVD have shown contradictory results. In some histological studies, mast-cell-predominant inflammation was demonstrated ^10, 13, 38^, while others reported inflammation without mast cell predominance ^30^ or the absence of inflammatory cells ^12, 31^.

Assays for pro-inflammatory molecules in vulvovaginal samples have also shown inconsistent results, with some studies reporting an elevation in the pro-inflammatory cytokines interleukin (IL)-1b and tumor necrosis factor (TNF)-α ^39^ and increased levels of the neurokine CGRP ^14^, while others found lower levels of TNF-α and similar levels of IL-1b among patients and controls ^40^. Nitric oxide synthase and cyclooxygenase 2 were not upregulated in vestibular mucosa biopsies from PVD patients ^34^, which is in opposition to ongoing cell-mediated inflammation ^26^. Laser Doppler perfusion imaging showed increased superficial blood flow in the mucosa, further supporting the neurogenic inflammation theory ^35^.

Levels of systemic interferon (IFN)-α and IFN-γ were similar in PVD patients and controls ^41^. A systematic review by Chalmers *et al*. published recently ^42^ highlights the lack of a consistent inflammatory profile in PVD patients.

Vulvovaginal infections are frequently cited as an inciting inflammatory event triggering the development of PVD. Often, PVD patients report a history of recurrent vulvovaginal candidiasis (RVVC), and they frequently relate the onset of their symptoms to a symptomatic vaginal candidiasis. Whether this represents an accurate association or is mainly a misinterpretation of patients’ symptoms is not clear ^43^, as the history of RVVC has often been based on self-report and the presence of the yeast was not confirmed by culture. Nevertheless, various studies described findings associated with a possible deficient immune response that resulted in RVVC and subsequent development of PVD. It has been postulated that an inability to clear vulvovaginal infections and the resulting chronic inflammation may lead to PVD development.

Circulating natural killer cells, a predominant factor in vaginal defense against Candida infections, are significantly lower in PVD patients ^44^. Other observations imply a possible genetic variability causing a predisposition to RVVC ^45– 47^. Additionally, an increased cutaneous hypersensitivity to *Candida albicans* organisms was reported in women with PVD ^48^. In an RVVC mouse model ^49^, chronic vulvar pain and increased vulvar innervations were also observed.

Foster *et al*. reported that vulvar fibroblasts produce high levels of IL-6, IL-8, and prostaglandin E2 (PGE2) following stimulation by irritants in both women with PVD and controls ^50, 51^. Vestibular fibroblasts released elevated levels of IL-6 and PGE2 compared to fibroblasts isolated from non-painful vulvar sites. Furthermore, pro-inflammatory mediator production was elevated in PVD fibroblasts compared with controls. Similar findings were reported when fibroblasts were challenged with live yeast species ^51^, and the response was highly predictive of clinically measured pain thresholds. The authors concluded that vulvar tissue of women with and without PVD can be differentiated by the degree of naturally occurring inflammation ^52^. They suggested that the vestibule of PVD patients is inherently more sensitive to yeast and that even a subclinical infection may trigger a maladaptive immune response in these fibroblasts.

In recently published work, the same group evaluated the signaling pathways involved in the recognition of yeast components that are present during chronic infection ^53^. They found that vestibular fibroblasts from PVD patients express elevated levels of Dectin-1, a surface receptor that binds *C. albicans*. They also showed that blocking the function or expression of Dectin-1 *in vitro* resulted in a significant decrease in IL-6 and PGE2 production.

In summary, our understanding of inflammation as a possible contributing factor to PVD development is still evolving. Recent studies have revisited this potential origin, suggesting that inflammation is likely to play a role in PVD.

## Hormonal factors

A possible correlation between hormonal contraception preparations (HCs) and PVD has been investigated primarily in epidemiological studies. Several reports have demonstrated that HCs increase the risk of developing PVD2 ^54– 57^. Results showed a 6.6 relative risk of PVD for ever-users of HCs compared to non-users, rising with increased duration of use (at least up to 2–4 years of use) and first use at a young age (<16 years) ^56^. The relative risk was higher when the product used was progestogenic and androgenic but low in estrogenic potency ^56^. The use of low-estrogen HCs (≤20 mcg ethinylestradiol) was significantly more common in women with PVD than in the general population of HC users ^57^. Burrows and Goldstein ^58^ described a case series of 50 women who developed PVD while on HCs and who were successfully treated with topical estradiol and testosterone. However, these findings were not confirmed in two epidemiologic studies ^59, 60^.

The effect of HCs on vestibular mucosa is probably multifactorial. HCs modify the morphological pattern of the vestibular mucosa, with the appearance of shallow and sparse dermal papillae ^61^. This effect may contribute to the decreased mechanical pain thresholds reported in healthy women using HCs ^62^. These morphological alterations may influence mechanical properties by thinning the epithelium and causing nerve endings to become more superficially located, thus altering the transduction of mechanical pressure to the receptors without affecting nerve fibers.

HCs can also affect the vestibular epithelium through interaction with hormone receptors or alteration of receptor expression ^63– 65^. Results from studies investigating the expression of estrogen receptor α (ERα) in PVD patients are contradictory. Eva *et al*. ^65^ reported a decrease in vestibular ERα in women with PVD, while Johannesson *et al*. ^63^ reported an increased amount of vestibular ERα in patients who were past HC users compared to controls. Healthy HC users displayed a higher amount of ERβ in the vestibular stroma compared with healthy non-users ^64^.

Another possible mechanism involves alteration of serum hormone levels. In women on HCs, there is suppression of ovarian testosterone production, reduced ovarian estradiol, and increased sex hormone binding globulin (SHBG) synthesis. This combination leads to low calculated free testosterone and low estradiol. Decreased estradiol may further contribute to vestibular atrophy found in patients with PVD2 ^66^, causing introital pain. It was also found that androgen receptors (ARs) are significantly lowered in vestibular tissue and cells of the minor vestibular glands in HC users ^67^. Additionally, some HCs contain synthetic progestins that act as testosterone antagonists at the AR ^68^. Goldstein *et al*. ^69^ identified a genetic polymorphism in the AR in PVD patients and concluded that an inefficient AR combined with lowered free testosterone predisposes to PVD. This has not been confirmed.

Other potential mechanisms refer to estrogen and progesterone as endogenous pain modulators ^70^. Endogenous pain modulation was found to be less effective in HC users ^71^, and a persistent genital hyperinnervation via a direct effect of synthetic progesterone on unmyelinated sensory nociceptor neurons has been described in animal models ^24^.

In summary, the association between HC use and the development of PVD is possible. The actual prevalence and susceptibility factors remain incompletely elucidated and it is thus not possible to make clinical recommendations. It is also not clear whether termination of HC usage alone can reverse PVD and which additional treatments may also be necessary.

## Pelvic floor muscle dysfunction

The muscles of the pelvic floor (PFM) consist of three layers: superficial, intermediate, and the deeper muscles known collectively as the “levator ani”. The PFM participate in multiple activities, including mechanical support of the pelvic organs, trunk stability and mobility, defecation, urination, closure of the urinary and anal orifices, and enhancement of sexual pleasure ^72^.

PFM dysfunction (PFMD) generally refers to disorders of laxity (hypotonus) or to muscles’ overactivity (hypertonicity). PFMD can result from various causes, including musculoskeletal factors, inflammation, trauma, pregnancy, vaginal delivery, abdominal or pelvic surgery, neuropathic pain, past or current psychological, physical, and/or sexual abuse, and anxiety in addition to a variety of other factors. Pelvic pain disorders and dyspareunia are usually associated with pelvic floor overactivity ^72^. Muscle contraction in response to pain has been referred to as guarding, while persistent states of muscle overactivity have been described as spasticity or hypertonus and are more frequently associated with amplification in neurological tone rather than as a response to pain ^72^.

It has long been recognized that PVD is often associated with some degree of PFMD, such as elevated resting tone ^73– 75^, increased contractile responses to painful stimuli ^75^, decreased flexibility, lower relaxation capacity ^73– 75^, lower pain thresholds ^76^, and lesser strength ^73^ compared with controls. Reissing *et al*. reported that 90% of the women in their study diagnosed with PVD demonstrated PFMD ^73^.

The mechanisms associating PFM hypertonus and PVD are not fully understood. Vaginal closure is assisted by the bulbospongiosus and puborectalis muscles, as well as activation of the levator ani ^72^. Reissing *et al*. ^73^ demonstrated that PVD patients displayed considerably higher PFM tone in the superficial layer, and these findings were less remarkable at the deeper PFM layers. They concluded that the absence of generalized hypertonicity may indicate that PFM hypertonus could result from, rather than cause, PVD. They hypothesized that muscle tension starts as a protective response to vestibular pain, and this response later results in increased resting muscle tone ^73– 75^. Increased PFM contraction causes an enhancement of pressure on the vestibule during penetration, further mounting both pain and the protective guarding reaction. Hypertonicity may, consequently, act to maintain as well as to exacerbate PVD. Recently, transperineal three-dimensional real-time ultrasound imaging has been used to investigate the morphology and function of the PFM in PVD patients ^77, 78^. Because this method does not involve vaginal penetration (as with intravaginal palpation and EMG assessment) and is therefore pain-free, it has the advantage of limiting bias caused by the participant’s pain and anxiety during the procedure ^79^. In accordance with previous studies, ultrasound imaging showed higher PFM tone and lesser contractile capacities among PVD patients ^77– 79^. These findings suggest that PVD patients display PFM impairments which are not limited to a defense reaction but are rather chronic ^78^.

Alternatively, it has been suggested that PFMD may result from a chronic inflammatory process in the vestibular mucosa. According to this theory, mucosal inflammation or trauma may induce hypersensitivity and contraction of the underlying PFM ^80^. This may contribute to sensitization of muscle pain receptors, which, in turn, through a development of a central sensitization process, diminish sensory pain thresholds. This hypothesis suggests a “vicious cycle” of inflammation and further muscle contraction ^36, 81, 82^.

Another hypothesis proposes the opposite pathway, i.e. an underlying PFMD may act as an initiator of sensory changes in susceptible mucosa ^36^. The mechanism suggested to be involved in such a pathway is an abnormal neuropathic output state due to changes in the peripheral and central nervous systems secondary to noxious stimuli over a prolonged period of time ^83^. It was also hypothesized that trigger points within the PFM may refer pain to the region of the vestibule ^73, 79^ or that hypertonicity of the muscles that insert at the posterior vestibule (pubococcygeus, puborectalis, and superficial transverse perineum) can lead to allodynia in the posterior vestibule due to altered neurodynamics and neural and tissue hypoxia ^20^.

In summary, the PFMD commonly documented in PVD patients, although clinically similar, could, in fact, be driven by distinctly different pathophysiological processes. These aforementioned theories have yet to be fully substantiated at the foundational level. Understanding the pathophysiological pathways will enable clinicians to target therapies to specific causes of vulvar pain, mucosal or muscular, as well as to recommend direct physical therapy regimens according to the type of pain and its contributors ^84^.

## Central pain mechanisms

Among etiological factors participating in PVD development, the alteration of central pain perception has also been suggested based on various observations. Comorbidity of PVD with other pain syndromes is often reported, most notably with irritable bowel syndrome, fibromyalgia, interstitial cystitis, and orofacial pain ^85^. Patients often suffer from other bodily pain and have lower pain thresholds in regions remote from the vestibule ^6^. In addition, an increased number of painful tender points were reported ^86^ and enhancement of a post-capsaicin pain response was found ^87^, suggesting central sensitization. Moreover, functional magnetic resonance imaging, performed during painful vestibular pressure, revealed similar activation in cerebral pain centers as in other chronic pain conditions ^88^, and grey matter density was increased in pain modulatory and stress-related areas of the brain compared to controls ^89^.

Taken together, these findings indicate that an abnormal feature of pain amplification may exist in PVD patients. This pain sensitivity may be attributed to either the chronicity of pain (central sensitization) ^6, 90^ or alternatively can be viewed as a reflection of an intrinsic defect in mechanisms of pain regulation ^91, 92^. As PVD1 patients exhibit greater sensitivity to thermal pain in comparison to women with PVD2, it was suggested that subgroups of PVD patients are different with regard to extragenital pain thresholds, implying differences in underlying pathophysiological mechanisms ^93^. Consideration of the contribution of central pain regulatory mechanisms may help to explain the variations in the clinical presentation and treatment outcomes in subgroups of PVD patients ^36, 93^.

In addition to comorbidity with pain syndromes, the incidence of depression, anxiety, stress, somatization, phobic symptoms, and catastrophizing is higher in PVD patients than in control women ^94^. These psychosocial and emotional factors influence the expression, clinical presentation, and response to therapy. It is unknown whether these disorders have a causal role in PVD, whether they represent a response to a chronic disabling medical disorder, or if they share a dysfunction in central-regulatory systems with chronic pain and, as such, coexist ^95^.

## Genetic factors

Several studies point to a possible genetic involvement in PVD based on several mechanisms:

- Polymorphisms in genes regulating the inflammatory response: variability in the DNA sequence of genes regulating immune recognition or magnitude and direction of the response to infections may explain an altered inflammatory response in patients with PVD (see above). Such genetic variations, termed “polymorphisms”, were described with regard to the IL-1 receptor antagonist ^96, 97^, IL-1b ^98^, mannose-binding lectin (MBL) ^45, 46^, NALP-3 ^47^, and the melanocortin-1 receptor genes ^97^. These polymorphic genes were suggested to influence susceptibility to PVD, severity of symptoms, or both.
- Polymorphisms in genes associated with an increased sensitivity to pain: the serotonin receptor gene *5HT-2A* ^99^
- Polymorphisms in genes involved in the effect of hormonal changes caused by HCs ^69^

In addition to these case-control studies, a recent study by Morgan *et al*. ^100^ evaluated whether PVD is more common in female relatives of women diagnosed with PVD using population-based genealogy-coded data. They found that the relative risk of vestibulectomy was elevated in first-, second-, and third-degree relatives and concluded that this familial clustering supports a genetic predisposition for PVD and warrants further studies to identify the specific genes involved.

## Summary

Clinical observations and epidemiological studies emphasize the heterogeneity of vulvar pain syndromes. In addition, data from basic research suggest different mechanisms relevant to the division of women with PVD into subgroups. However, in clinical practice, the relative contributions of different triggering or persistent factors remain poorly understood, and current diagnostic criteria are based on highly subjective measures, while treatment proceeds on a trial-and-error approach. The result is that many forms of therapeutic interventions have been used, yet the evidence remains largely inconclusive, the response rate varies between 40 and 85%, and many women with PVD do not respond to any of the treatments ^5^. Inconclusive data regarding etiology, epidemiology, mucosal characteristics, and treatment results can be explained by grouping patients who in reality have different underlying conditions under a single diagnosis and then evaluating the efficacy of a single intervention that might be relevant to only one subset of patients. This can lead to an apparent lack of significance or effect due to dilution caused by the heterogeneous patient population.

Constructing an algorithm that may enable the allocation of patients into PVD subgroups will advance both basic science and clinical research and improve future medical management. Although a large research effort has been made in this area, only one such clinical algorithm has been suggested, distinguishing between four subtypes of PVD ^21, 101^: hormonally mediated PVD, hypertonic pelvic floor dysfunction, congenital neuroproliferative PVD, and acquired neuroproliferative PVD (secondary to inflammation), based on a patient’s history and physical examination. According to our personal experience, this classification provides better outcomes compared to the trial-and-error approach we used before (higher response rates as well as shortening the period for significant improvement). However, objective data are missing to support this algorithm.

Given the high incidence of PVD in the population and its huge effect on patients’ lives, there is a critical need to perform prospective, longitudinal studies that will allow better diagnostic criteria and subgrouping of patients that would lead to improvements in our understanding of PVD and its treatment.
